# Prognostic value of the atherogenic index of plasma in patients with acute coronary syndrome without standard modifiable risk factors: a machine learning-based cohort study

**DOI:** 10.3389/fcvm.2025.1681170

**Published:** 2025-10-07

**Authors:** Zheng Chen, Xiaoli Liu, Yan Sun, Dai Zhang, Yujing Cheng, Yujie Zhou

**Affiliations:** Beijing Key Laboratory of Precision Medicine of Coronary Atherosclerotic Disease, Department of Cardiology, Beijing Anzhen Hospital, Beijing Institute of Heart Lung and Blood Vessel Disease, Clinical Center for Coronary Heart Disease, Capital Medical University, Beijing, China

**Keywords:** atherogenic index of plasma, standard modifiable cardiovascular risk factors, acute coronary syndrome, major adverse cardiovascular and cerebrovascular events, prognosis

## Abstract

**Background:**

Patients without any standard modifiable cardiovascular risk factors (SMuRF-less) who develop acute coronary syndrome (ACS), tend to have poor outcomes. However, the prognostic value of atherogenic index of plasma (AIP) in these patients is unclear. Therefore, we investigated the association between AIP and adverse outcomes in SMuRF-less patients with ACS.

**Methods:**

This study retrospectively enrolled 722 SMuRF-less patients with ACS receiving percutaneous coronary intervention (PCI) at Beijing Anzhen Hospital from March 2017 to March 2018. Three patient-groups were formed using AIP tertiles. The primary outcome, major adverse cardiovascular and cerebrovascular events (MACCE), was a composite of all-cause mortality, non-fatal myocardial infarction (MI), unplanned revascularization, and non-fatal ischemic stroke. Association between AIP levels and MACCE risk was examined using restricted cubic spline (RCS) analysis. Prognostic value of AIP levels for MACCE was assessed using multivariable Cox regression models and machine learning approaches.

**Results:**

During follow-up of the 722 patients (median age, 60 years [interquartile range, 53–67]; female, 29.8%; median follow-up duration, 59 months), 168 (23.3%) developed MACCE. The RCS results showed linear association of progressively increasing MACCE risk with increasing AIP levels. In multivariable Cox regression analysis, significantly higher MACCE risk occurred with the highest AIP tertile than with the lowest (hazard ratio [HR] 2.03, 95% confidence interval [CI]: 1.34–3.08; *P* < 0.001). Elevated AIP level was associated with higher risks of all-cause death (HR: 3.49, 95% CI: 1.09–11.23; *P* = 0.036); non-fatal MI (HR: 3.02, 95% CI: 1.08–8.48; *P* = 0.035); and unplanned revascularization (HR: 2.18, 95% CI: 1.34–3.52; *P* < 0.001). As a continuous variable, higher AIP levels were significantly associated with increased risks of MACCE (HR: 2.95, 95% CI: 1.74–4.98; *P* < 0.001), all-cause mortality (HR: 6.80, 95% CI: 1.85–24.96; *P* = 0.003), non-fatal myocardial infarction (HR: 3.58, 95% CI: 1.08–11.86; *P* = 0.037), and unplanned revascularization (HR: 2.84, 95% CI: 1.55–5.19; *P* < 0.001). Machine-learning models incorporating AIP levels improved outcome prediction. At 48 months, the gradient boosting machine model achieved the highest AUC (0.796; 95% CI: 0.703–0.889), while complementary assessments showed that the random survival forest model provided the greatest net clinical benefit and demonstrated excellent calibration.

**Conclusion:**

Among SMuRF-less patients with ACS undergoing PCI, AIP level was identified as an independent predictor of clinical prognosis.

## Graphical Abstract

**Figure:**
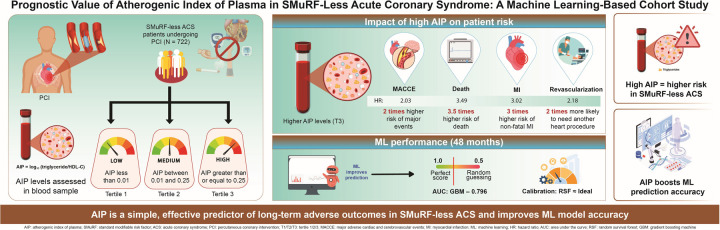

## 1 Introduction

Coronary heart disease, the most prevalent cardiovascular illness worldwide, continues to be a major contributor to mortality, disability, and healthcare burden (1). Standard modifiable cardiovascular risk factor (SMuRF) less patients refers to those without conventional modifiable risk factors, such as hypertension, diabetes, hyperlipidemia, or smoking (2). However, in the absence of these standard risk factors, individuals can still develop coronary heart disease (3). Evidence from large registry studies, including SWEDEHEART and CCC-ACS, suggests that patients with acute coronary syndrome (ACS) who lack standard modifiable risk factors (SMuRF-less) tend to experience worse prognoses compared with those carrying at least one SMuRF (4, 5). This highlights an urgent need for prognostic markers to identify high-risk SMuRF-less patients early, guide timely intervention, and ultimately improve their outcomes.

Growing evidence indicates that, beyond traditional risk factors, other mechanisms significantly influence the development of coronary heart disease. Insulin resistance (IR) and inflammatory states are among these important contributors (6–8). The atherogenic index of plasma (AIP) level, calculated as log₁₀(triglyceride/high density lipoprotein cholesterol [HDL-C]), serves as an integrated index based on the serum levels of triglyceride and HDL-C, and it correlates with both IR and systemic inflammation (9, 10). Previous studies have demonstrated that AIP level has prognostic value in various cardiovascular conditions. AIP level could predict worse outcomes in chronic coronary syndrome (CCS) and acute decompensated heart failure, and it is associated with the incidence of hypertension, carotid atherosclerosis, and ischemic stroke (11–16). A systematic review and meta-analysis including 20,833 patients with coronary artery disease also demonstrated significant association of higher AIP levels with increased risks of major adverse cardiovascular events, as well as cardiovascular death, myocardial infarction, and revascularization (17). However, to date, no study has specifically evaluated AIP level's prognostic relevance in SMuRF-less patients with ACS. Therefore, we aimed to investigate the association between AIP level and adverse outcomes in SMuRF-less patients with ACS undergoing percutaneous coronary intervention (PCI), to facilitate early risk identification and improve clinical prognosis.

## 2 Methods

### 2.1 Study population

**Figure 1:**
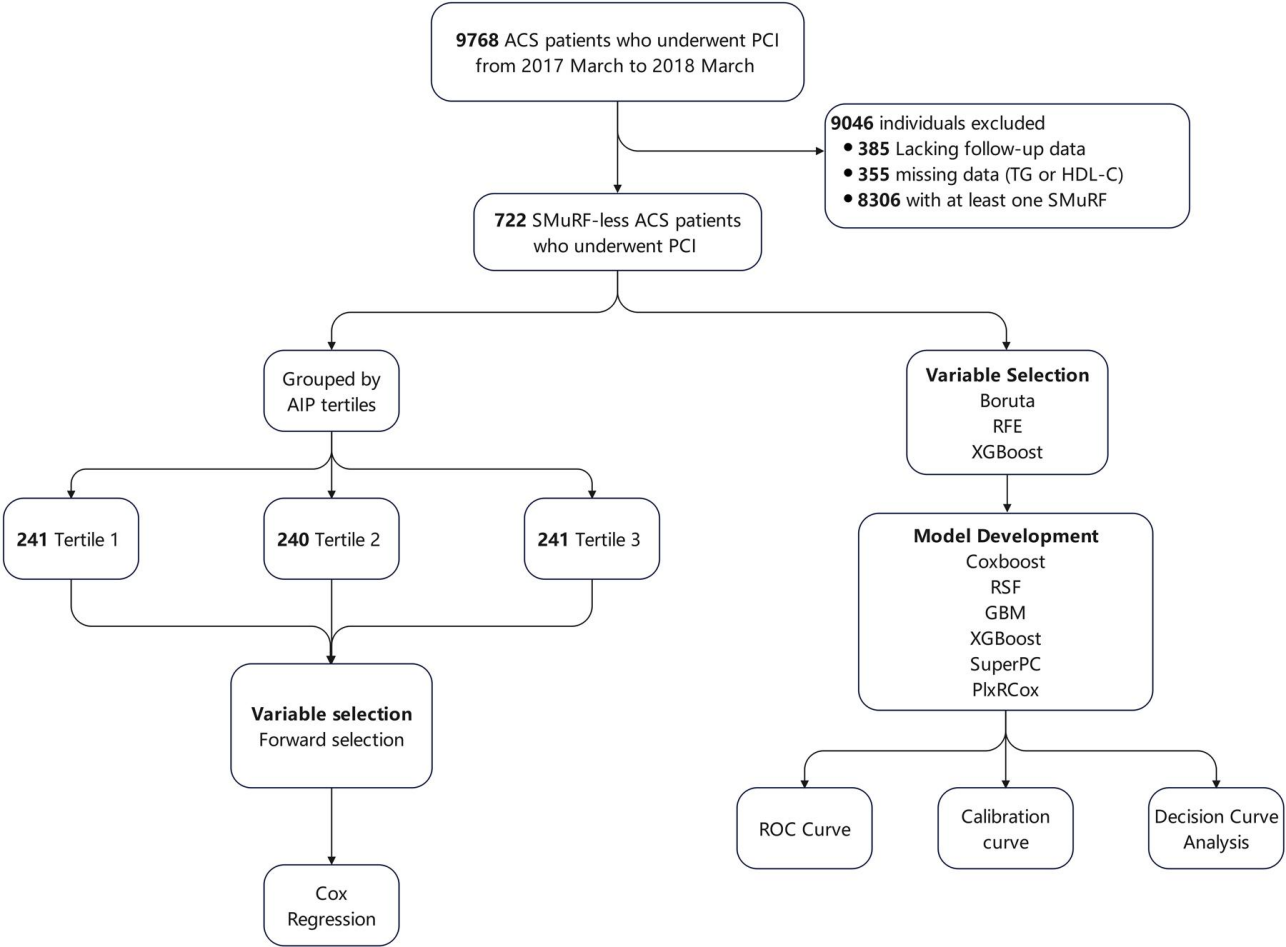
Study flowchart.

A total of 9,768 patients with ACS undergoing PCI were retrospectively enrolled at Beijing Anzhen Hospital from March 2017 to March 2018. ACS encompassed ST-segment elevation myocardial infarction (STEMI), non-ST-segment elevation myocardial infarction (NSTEMI), and unstable angina (UA). ACS diagnostic criteria complied with the American College of Cardiology/American Heart Association guidelines (18). Inclusion criteria for this study were: (1) diagnosed with ACS; (2) underwent PCI; and (3) SMuRF-less. Patients who (1) lacked follow-up data, (2) had missing data on triglyceride or HDL-C, and (3) had one or more SMuRFs, were excluded. The definitions of SMuRFs (hypertension, diabetes, hyperlipidemia, and current smoking) are provided in Section 2.2. Of the screened patients, 9,046 were excluded: 385 due to the lack of follow-up data, 355 due to missing data on triglyceride or HDL-C concentrations, and 8,306 due to having one or more SMuRFs. Finally, 722 SMuRF-less ACS patients were included in the analysis. The flow diagram of patient inclusion is presented in Figure 1. Ethical approval was granted by the Institutional Review Board of Beijing Anzhen Hospital (No. 2025159X).

### 2.2 Definition of SMuRF

SMuRFs in this study included hypertension, diabetes, hyperlipidemia, and current smoking (19). They were defined as follows:

• **Hypertension:** history of hypertension, or use of antihypertensive agents prior to admission, or a new diagnosis of hypertension during the hospitalization.
• **Diabetes:** a previous diagnosis of diabetes, use of glucose-lowering medications prior to hospitalization, or a new diagnosis of diabetes during the index hospitalization.
• **Hyperlipidemia:** known history of hyperlipidemia, use of lipid-lowering therapy before admission, or baseline low-density lipoprotein cholesterol (LDL-C) level ≥ 3.5 mmol/L or total cholesterol (TC) ≥5.5 mmol/L.
• **Current smoking:** defined as ongoing smoking within 30 days before admission.

### 2.3 Data collection

The following were collected: demographic data (age, sex, height, and weight); vital signs (systolic [SBP] and diastolic blood pressure [DBP]); heart rate; ACS classification (UA, NSTEMI, or STEMI); past medical history (of prior PCI, coronary artery bypass grafting [CABG], carotid artery stenosis, and peripheral arterial disease); laboratory parameters (leukocyte, hemoglobin, platelet count, albumin, alanine transaminase [ALT], aspartate transaminase [AST], TC, LDL-C, HDL-C, triglyceride, creatinine, sodium, potassium, fasting blood glucose [FBG], hemoglobin A1c [HbA1c], and B-type natriuretic peptide [BNP]); left ventricular ejection fraction (LVEF); medications (aspirin, clopidogrel, ticagrelor, angiotensin-converting enzyme inhibitors [ACEIs], angiotensin II receptor blockers [ARBs], statins, and beta blockers); angiographic results (triple vessel disease, left main, ostial disease, bifurcation lesions, chronic total occlusion [CTO], calcification, diffuse lesion, in-stent restenosis); and PCI outcomes (target vessel numbers, target vessel distribution, number of stents, stent diameter, and stent length).

Body mass index (BMI) was calculated as weight in kilograms divided by the square of height in meters. Triglyceride levels were measured using the glycerol-3-phosphate oxidase–peroxidase enzymatic method and HDL-C by enzymatic colorimetry. AIP level was calculated from the first available triglyceride and HDL-C values at admission using the formula: AIP = log_10_(triglyceride/HDL-C) (20).

### 2.4 Patient grouping according to AIP tertiles and the study endpoints

Based on the AIP tertiles: tertile 1 (AIP < 0.01); tertile 2 (0.01 ≤ AIP < 0.25); and tertile 3 (AIP ≥ 0.25), the patients were categorized into three groups. The primary endpoint was major adverse cardiovascular or cerebrovascular events (MACCE), and secondary endpoints were the individual MACCE components. MACCE was defined as the composite of all-cause mortality, non-fatal myocardial infarction (MI), unplanned revascularization, and non-fatal stroke (21). Non-fatal MI was diagnosed using the Fourth Universal Definition of MI (22), while non-fatal stroke was based on imaging (computed tomography or magnetic resonance imaging) combined with evidence of neurological deficits (23). Unplanned revascularization was defined as rehospitalization for recurrent angina followed by unplanned PCI or CABG. Follow-up was by trained personnel at 1, 6, 12, 36, and 60 months post-discharge during outpatient visits or by telephone interviews, during which the occurrence and timing of endpoint events were documented.

### 2.5 Statistical analysis

Missing data on all variables, which were <10%, were imputed using the k-nearest neighbor method (k = 5, Euclidean distance) after Min-Max scaling. To prevent information leakage, imputation was performed within the training set, and the fitted model was subsequently applied to the testing set. The proportion of missing data for each variable is provided in Supplementary Table S1. As the missing rate was <10% on all variables, the potential impact of imputation on the results is expected to be minimal. Baseline variables were summarized according to their distribution. Normally distributed continuous data are presented as mean ± standard deviation and compared across groups using one-way analysis of variance. Non-normally distributed variables are expressed as median (interquartile range) and analyzed using the Kruskal–Wallis test. Categorical variables are described using counts and percentages, with intergroup differences assessed by the chi-square test. Kaplan–Meier survival analysis and the log-rank test were used to assess differences in event-free survival among groups. Restricted cubic spline (RCS) curves with four knots placed at the 5th, 35th, 65th, and 95th percentiles of the AIP distribution were used to assess the dose–response association between AIP level and clinical outcomes. Cox proportional hazards regression was used to estimate hazard ratio (HR) and 95% confidence interval (CI) of the risk of the endpoints. The selection of covariates in the final multivariable model was based on both clinical relevance and univariate associations with the outcomes. Specifically, prior studies have demonstrated that age, sex, BMI, albumin, ACEI/ARB use, *β*-blocker use, CTO, and history of CABG are significantly associated with prognosis in ACS patients; therefore, these were prespecified as confounders (18, 24–29). In addition, creatinine, FBG, and leukocyte count showed significant univariate associations with the outcomes and were thus included. Candidate variables with a *P* value <0.05 in univariate analyses were considered for inclusion in the multivariable model. In Model 1, no adjustments were made. Model 2 was adjusted for age, sex, and BMI. Model 3 was further adjusted for history of CABG; leukocyte count; albumin; creatinine; FBG; use of ACEI/ARBs and beta blockers; and presence of CTO lesions.

Patients were also stratified into subgroups by age, sex, BMI, ACS classification, LDL-C level, and LVEF, in subgroup analyses, to determine whether these variables influenced the association between AIP and MACCE. For machine learning analysis, a correlation heatmap was first generated to detect multicollinearity. For pairs of variables with an absolute correlation >0.8, one of the correlated variables was excluded to avoid collinearity. The dataset was randomly split into training (70%, *n* = 506) and testing (30%, *n* = 216) cohorts. Three feature selection methods: Boruta algorithm, recursive feature elimination (RFE), and XGBoost importance ranking, were used to identify important variables. Six algorithms—CoxBoost, random survival forest (RSF), gradient boosting machine (GBM), XGBoost, supervised principal components (SuperPC), and partial least squares regression for Cox models (plsRcox)—were used to construct predictive models. The primary criterion for model performance comparison was the area under the curve (AUC) from the time-dependent receiver operating characteristic (ROC) analysis, given its widespread use and interpretability in clinical prediction models. Calibration plots and decision curve analysis (DCA) were additionally applied as complementary assessments to provide a more comprehensive evaluation of model performance. Two-tailed *P* < 0.05 was considered significant. Data analysis was performed using R (version 4.5.0, R Foundation for Statistical Computing, Vienna, Austria).

## 3 Results

### 3.1 Clinical and procedural characteristics of the patients

A total of 722 SMuRF-less patients with ACS were included in the analysis, in a median follow-up of 59 months. Tertile-based grouping was performed according to AIP values. Table 1 summarizes the baseline clinical and procedural characteristics. The patients median age was 60 years (interquartile range: 53–67), and 215 (29.8%) were female. Compared with the lowest tertile, patients in the highest AIP tertile had higher BMI; heart rate; leukocyte count; levels of ALT, TC, triglyceride, and creatinine; FBG; use of ACEI/ARBs; prevalence of CTO lesions; and proportion of target vessel right coronary artery (*P* < 0.05 for all comparisons). Meanwhile, they showed lower age and HDL-C levels (*P* < 0.05 for all comparisons). No significant differences were observed in the remaining variables among the groups.

**Table 1:** Baseline clinical and procedural characteristics by AIP tertiles.

Variables	Grouped by AIP tertiles				
	Overall (*N* = 722)	Tertile 1 (*n* = 241)	Tertile 2 (*n* = 240)	Tertile 3 (*n* = 241)	P-value
Age, years	60 (53, 67)	62 (55, 68)	61.5 (54, 68)	58 (51, 65)	0.002
Female, *n* (%)	215 (29.8)	69 (28.6)	73 (30.4)	73 (30.3)	0.892
BMI, kg/m^2^	25.5 (23.5, 27.6)	24.5 (22.6, 26.8)	25.5 (23.5, 27.6)	26.3 (24.3, 28.3)	<0.001
SBP, mmHg	124 (115, 131)	125 (115, 132)	124 (115, 130)	123 (115, 131)	0.454
DBP, mmHg	75 (69, 80)	75 (70, 80)	75 (68, 80)	75 (69, 81)	0.789
Heart rate, beat/min	70 (65, 78)	70 (65, 76)	70 (66, 76)	72 (66, 79)	0.029
ACS classification, *n* (%)					0.374
UA	546 (75.6)	190 (78.8)	184 (76.7)	172 (71.4)	
NSTEMI	90 (12.5)	26 (10.8)	27 (11.3)	37 (15.4)	
STEMI	86 (11.9)	25 (10.4)	29 (12.1)	32 (13.3)	
Histories, *n* (%)					
Prior PCI	194 (26.9)	66 (27.4)	61 (25.4)	67 (27.8)	0.820
Prior CABG	13 (1.8)	3 (1.2)	4 (1.7)	6 (2.5)	0.579
Carotid artery stenosis	32 (4.4)	13 (5.4)	10 (4.2)	9 (3.7)	0.656
Peripheral artery disease	7 (1)	1 (0.4)	2 (0.8)	4 (1.7)	0.309
Laboratory parameters					0.365
Leukocyte, ×10^9^/L	6.7 (5.66, 8.02)	6.33 (5.23, 7.44)	6.71 (5.70, 8.10)	7.04 (5.90, 8.49)	<0.001
Hemoglobin, g/L	139 (126, 150)	137 (126, 147)	140 (127, 151)	139 (126, 148)	0.400
Platelets, ×10^9^/L	221 (186, 256)	217 (183, 251)	220 (187, 256)	223 (186, 267)	0.371
Albumin, g/L	43.0 ± 3.4	43.3 ± 3.3	42.8 ± 3.4	42.8 ± 3.7	0.219
ALT, U/L	23 (16, 32)	20 (15, 29)	25 (18, 32)	24 (17, 34)	<0.001
AST, U/L	22 (19, 28)	22 (18, 27)	23 (19, 28)	22 (18, 27)	0.090
TC, mmol/L	3.94 (3.47, 4.41)	3.74 (3.29, 4.20)	3.74 (3.32, 4.25)	4.31 (3.89, 4.68)	<0.001
LDL-C, mmol/L	2.04 (1.7, 2.46)	2.03 (1.70, 2.50)	2.01 (1.71, 2.43)	2.06 (1.69, 2.44)	0.9682
HDL-C, mmol/L	1.06 (0.91, 1.22)	1.23 (1.08, 1.43)	1.02 (0.90, 1.15)	0.95 (0.83, 1.07)	<0.001
TG, mmol/L	1.38 (0.96, 2.12)	0.88 (0.73, 1.02)	1.38 (1.19, 1.63)	2.42 (2.08, 2.96)	<0.001
Creatinine, μmol/L	71 (61.02, 80.5)	68.9 (59.4, 78.7)	69.7 (60.5, 79.0)	74.0 (64.8, 83.6)	0.001
Na, mmol/L	139.3 (137.8, 141)	139.3 (137.6, 141.2)	139.6 (138.1, 141.0)	139.2 (137.9, 140.9)	0.670
K, mmol/L	4 (3.84, 4.23)	4 (3.85, 4.23)	4 (3.86, 4.23)	3.99 (3.82, 4.20)	0.526
FBG, mmol/L	5.39 (5.01, 5.92)	5.38 (5.01, 5.96)	5.31 (4.95, 5.76)	5.49 (5.10, 6.00)	0.030
HbA1C, %	5.8 (5.5, 6.1)	5.8 (5.5, 6)	5.8 (5.6, 6.1)	5.7 (5.5, 6)	0.216
BNP, pg/ml	47.5 (20, 94)	42.0 (19.0, 90.0)	52.0 (21.0, 101.5)	47.0 (21.0, 86.0)	0.303
LVEF, %	64 (60, 66)	64 (60, 66)	63 (59, 66)	63 (60, 66)	0.738
Medications, *n* (%)					
Aspirin	709 (98.2)	237 (98.3)	234 (97.5)	238 (98.8)	0.574
Clopidogrel	599 (83)	209 (86.7)	194 (80.8)	196 (81.3)	0.162
Ticagrelor	117 (16.2)	30 (12.4)	43 (17.9)	44 (18.3)	0.152
Statins	653 (90.4)	225 (93.4)	215 (89.6)	213 (88.4)	0.152
ACEI/ARBs	82 (11.4)	21 (8.7)	19 (7.9)	42 (17.4)	0.001
Beta blockers	428 (59.3)	135 (56.0)	136 (56.7)	157 (65.1)	0.075
Angiographic results, *n* (%)					
Triple vessel disease	152 (21.1)	52 (21.6)	52 (21.7)	48 (19.9)	0.869
Left main	76 (10.5)	27 (11.2)	25 (10.4)	24 (10.0)	0.904
Ostial disease	107 (14.8)	31 (12.9)	37 (15.4)	39 (16.2)	0.562
bifurcation lesions	86 (11.9)	31 (12.9)	34 (14.2)	21 (8.7)	0.156
CTO	153 (21.2)	40 (16.6)	51 (21.3)	62 (25.7)	0.049
Calcification	103 (14.3)	41 (17.0)	31 (12.9)	31 (12.9)	0.328
Diffuse lesion	275 (38.1)	80 (33.2)	97 (40.4)	98 (40.7)	0.159
In-stent restenosis	24 (3.3)	8 (3.3)	7 (2.9)	9 (3.7)	0.882
PCI outcomes					
Target vessel numbers, *n* (%)					0.816
1	588 (81.4)	199 (82.6)	195 (81.3)	194 (80.5)	
2	118 (16.3)	36 (14.9)	40 (16.7)	42 (17.4)	
3	15 (2.1)	6 (2.5)	5 (2.1)	4 (1.7)	
4	1 (0.1)	0	0	1 (0.4)	
Target vessel distribution, *n* (%)					
Left main	49 (6.8)	18 (7.5)	15 (6.3)	16 (6.6)	0.863
LAD	399 (55.3)	147 (61.0)	128 (53.3)	124 (51.5)	0.083
LCX	167 (23.1)	54 (22.4)	52 (21.7)	61 (25.3)	0.605
RCA	258 (35.7)	70 (29.0)	95 (39.6)	93 (38.6)	0.029
Number of stents, n	1 (1, 2)	1 (1, 2)	1 (1, 2)	1 (1, 2)	0.348
Stent diameter, mm	3 (2.63, 3.38)	3 (2.7, 3.5)	3 (2.63, 3.31)	3 (2.63, 3.38)	0.721
Stent length, mm	33 (20, 54)	30(20, 48)	33(20, 56)	33(20, 56)	0.548
AIP	0.14 ± 0.29	−0.17 ± 0.14	0.13 ± 0.07	0.44 ± 0.18	<0.001

ACEI, angiotensin converting enzyme inhibitor; ACS, acute coronary syndrome; AIP, atherogenic index of plasma; ALT, alanine transaminase; ARB, angiotensin II receptor blocker; AST, aspartate transaminase; BMI, body mass index; BNP, B-type natriuretic peptide; CABG, coronary artery bypass grafting; CTO, chronic total occlusion; DBP, diastolic blood pressure; FBG, fasting blood glucose; HbA1c, hemoglobin A1c; HDL-C, high-density lipoprotein cholesterol; LAD, left anterior descending artery; LCX, left circumflex artery; LDL-C, low-density lipoprotein cholesterol; LVEF, left ventricular ejection fraction; NSTEMI, non-ST-elevation myocardial infarction; PCI, percutaneous coronary intervention; RCA, right coronary artery; SBP, systolic blood pressure; STEMI, ST-elevation myocardial infarction; TC, total cholesterol; TG, triglyceride; UA, unstable angina.

### 3.2 Association between AIP level and adverse outcomes

During the follow-up, 168 (23.3%) patients experienced MACCE (Supplementary Table S2). Compared to the lower tertiles, patients in the highest AIP tertile had significantly higher incidence rates of MACCE (14.9% vs. 22.9% vs. 32.0%, *P* < 0.001); all-cause mortality (1.7% vs. 3.3% vs. 6.2%, *P* = 0.028); non-fatal myocardial infarction (2.1% vs. 3.8% vs. 7.1%, *P* = 0.023); and unplanned revascularization (10.8% vs. 17.0% vs. 24.9%, *P* < 0.001) whereas the incidence of non-fatal stroke did not differ significantly among groups (2.5% vs. 3.3% vs. 4.6%, *P* = 0.456).

**Figure 2:**
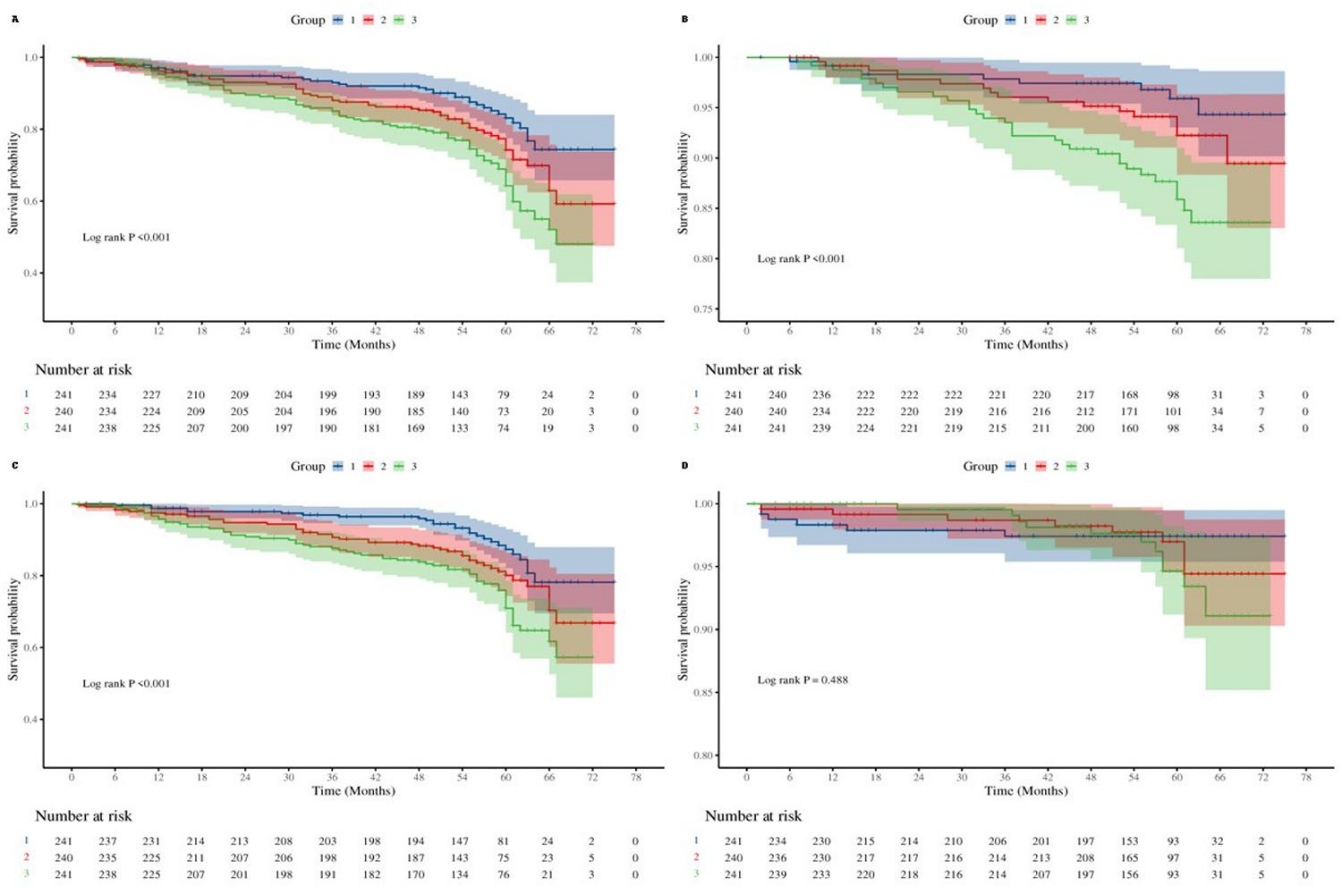
Kaplan–meier curves for outcomes by atherogenic index of plasma (AIP) tertile. **(A)** Major adverse cardiac and cerebrovascular events (MACCE) events. **(B)** All-cause mortality + non-fatal myocardial infarction. **(C)** Unplanned revascularization. **(D)** Non-fatal stroke.

Event-free survival for MACCE and its components was significantly lower in higher AIP tertiles, as demonstrated by Kaplan–Meier curves (log-rank *P* < 0.05 for all comparisons; Figure 2).

**Figure 3:**
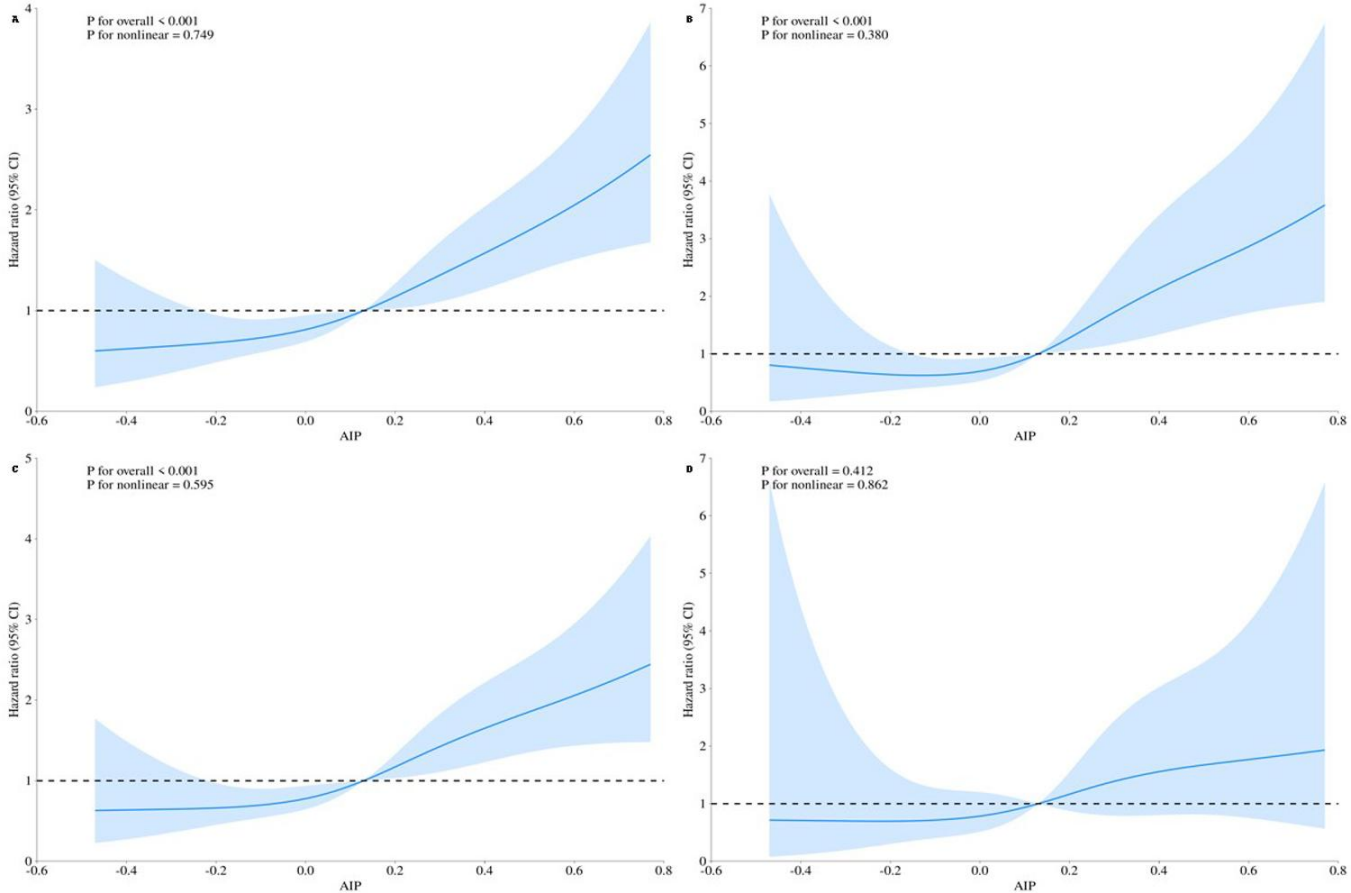
Restricted cubic spline curves for the outcomes according to the AIP level. The solid blue line represents the estimated hazard ratio, and the shaded area indicates the 95% confidence interval. **(A)** MACCE events. **(B)** All-cause mortality + non-fatal myocardial infarction. **(C)** Unplanned revascularization. **(D)** Non-fatal stroke.

RCS analyses (Figure 3) visually confirmed the relationships between AIP and each outcome. AIP was linearly associated with the risk of each outcome (nonlinearity tests *P* > 0.05), and an upward trend in AIP was associated with a corresponding rise in the risks of MACCE and its individual components (overall *P* < 0.05). The solid blue lines in Figure 3 represent the HRs, while the shaded areas indicate the 95% CIs.

### 3.3 Cox regression analysis

Cox regression models confirmed the associations between AIP and adverse outcomes (Table 2). In unadjusted analysis, compared to the reference group (tertile 1), tertile groups 2 and 3 HRs (95% CIs) for MACCE were 1.57 (1.03–2.38; *P* = 0.036) and 2.26 (1.52–3.36; *P* < 0.001), respectively. For all-cause death, tertile groups 2 and 3 HRs were 1.93 (0.58–6.40; *P* = 0.284) and 3.68 (1.22–11.08; *P* = 0.021), respectively. For non-fatal MI, HRs were 1.73 (0.58–5.17; *P* = 0.324) and 3.42 (1.26–9.26; *P* = 0.005), respectively. For unplanned revascularization, these were 1.70 (1.04–2.76; *P* = 0.034) and 2.47 (1.56–3.92; *P* < 0.001), respectively. Non-fatal stroke showed no significant differences between groups.

**Table 2:** Cox regression analysis of the association AIP tertiles with adverse outcomes.

Model	MACCE		All-cause mortality		Non-fatal myocardial infarction		Unplanned revascularization		Non-fatal stroke	
	HR (95% CI)	P-value	HR (95% CI)	P-value	HR (95% CI)	P-value	HR (95% CI)	P-value	HR (95% CI)	P-value
Model 1										
Tertile 1	Reference		Reference		Reference		Reference		Reference	
Tertile 2	1.57 (1.03–2.38)	0.036	1.93 (0.58–6.40)	0.284	1.73 (0.58–5.17)	0.324	1.70 (1.04–2.76)	0.034	1.28 (0.44–3.68)	0.650
Tertile 3	2.26 (1.52–3.36)	<0.001	3.68 (1.22–11.08)	0.021	3.42 (1.26–9.26)	0.016	2.47 (1.56–3.92)	<0.001	1.79 (0.66–4.83)	0.253
Continuous	3.95 (2.41–6.49)	<0.001	7.25 (2.59–20.29)	<0.001	4.54 (1.56–13.18)	0.005	3.96 (2.24–6.98)	<0.001	2.98 (0.82–10.87)	0.097
Model 2										
Tertile 1	Reference		Reference		Reference		Reference		Reference	
Tertile 2	1.57 (1.03–2.39)	0.036	1.99 (0.60–6.62)	0.261	1.74 (0.58–5.20)	0.320	1.70 (1.04–2.76)	0.033	1.28 (0.44–3.70)	0.646
Tertile 3	2.41 (1.61–3.60)	<0.001	5.20 (1.69–16.07)	0.004	3.27 (1.20–8.93)	0.021	2.55 (1.60–4.07)	<0.001	1.93 (0.70–5.30)	0.201
Continuous	4.39 (2.65–7.28)	<0.001	12.76 (4.15–39.20)	<0.001	4.52 (1.48–13.81)	0.008	4.18 (2.35–7.45)	<0.001	3.12 (0.89–10.95)	0.075
Model 3										
Tertile 1	Reference		Reference		Reference		Reference		Reference	
Tertile 2	1.56 (1.02–2.39)	0.041	1.76 (0.52–5.94)	0.360	1.69 (0.56–5.11)	0.350	1.68 (1.03–2.76)	0.038	1.28 (0.44–3.74)	0.650
Tertile 3	2.03 (1.34–3.08)	<0.001	3.49 (1.09–11.23)	0.036	3.02 (1.08–8.48)	0.035	2.18 (1.34–3.52)	<0.001	2.05 (0.72–5.84)	0.179
Continuous	2.95 (1.74–4.98)	<0.001	6.80 (1.85–24.96)	0.003	3.58 (1.08–11.86)	0.037	2.84 (1.55–5.19)	<0.001	3.90 (0.97–15.62)	0.055

AIP, atherogenic index of plasma; MACCE, major adverse cardiac and cerebrovascular events.

Model 1: unadjusted.

Model 2: adjusted for age, sex, and body mass index.

Model 3: adjusted for variables in Model 2 plus albumin, use of angiotensin-converting enzyme inhibitors or angiotensin II receptor blockers, beta blockers, history of coronary artery bypass grafting, chronic total occlusion lesion, creatinine, fasting blood glucose, and leukocyte count.

After adjusting for age, sex, and BMI (Model 2), similar results were observed: compared to the tertile 1 group, tertile 2 and 3 groups' HRs were 1.57 (1.03–2.39; *P* = 0.036) and 2.41 (1.61–3.60; *P* < 0.001) for MACCE; 1.99 (0.60–6.62; *P* = 0.261) and 5.20 (1.69–16.07; *P* = 0.004), for all-cause mortality; 1.74 (0.58–5.20; *P* = 0.320) and 3.27 (1.20–8.93; *P* = 0.021) for non-fatal MI; and 1.70 (1.04–2.76; *P* = 0.033) and 2.55 (1.60–4.07; *P* < 0.001) for unplanned revascularization, respectively. Non-fatal stroke remained non-significant across models.

Further multivariate adjustment in Model 3 (including history of CABG, leukocyte count, albumin level, creatinine level, FBG, ACEI/ARBs and beta blockers use, and CTO lesions) yielded consistent results: compared to the tertile 1 group, tertile 2 and 3 groups' adjusted HRs were 1.56 (1.02–2.39; *P* = 0.041) and 2.03 (1.34–3.08; *P* < 0.001) for MACCE; 1.76 (0.52–5.94; *P* = 0.360) and 3.49 (1.09–11.23; *P* = 0.036) for all-cause mortality; 1.69 (0.56–5.11; *P* = 0.350) and 3.02 (1.08–8.48; *P* = 0.035) for non-fatal MI; and 1.68 (1.03–2.76; *P* = 0.038) and 2.18 (1.34–3.52; *P* < 0.001) for unplanned revascularization, respectively. Again, non-fatal stroke did not differ significantly between groups in any model.

When AIP levels were analyzed as a continuous variable, we observed that each unit increase in AIP level was associated with an approximately 2.95-fold higher risk of MACCE (1.74–4.98; *P* < 0.001), a 6.80-fold higher risk of all-cause mortality (1.85–24.96; *P* = 0.003), a 3.58-fold higher risk of non-fatal myocardial infarction (1.08–11.86; *P* = 0.037), and a 2.84-fold higher risk of unplanned revascularization (1.55–5.19; *P* < 0.001). The association with non-fatal stroke showed a similar trend but did not reach statistical significance (0.97–15.62; *P* = 0.055).

### 3.4 Subgroup analysis results

**Figure 4:**
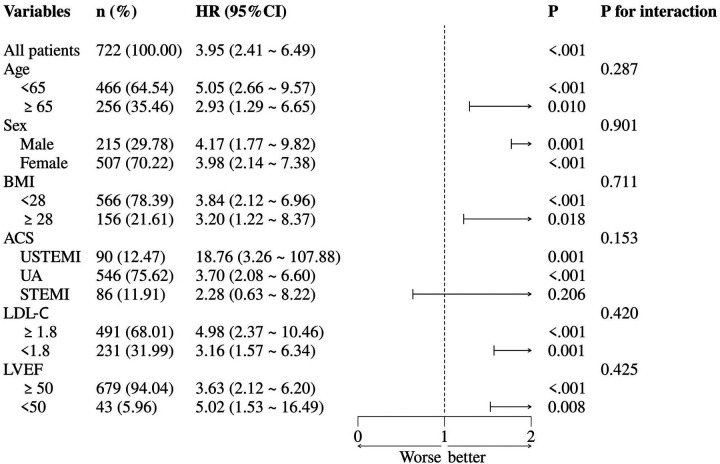
Subgroup analysis. ACS, acute coronary syndrome; BMI, body mass index; LDL-C, low-density lipoprotein cholesterol; LVEF, left ventricular ejection fraction; NSTEMI, non-ST-elevation myocardial infarction; STEMI, ST-elevation myocardial infarction; UA, unstable angina.

Patients were stratified by age, sex, BMI, ACS classification, LDL-C level, and LVEF for the subgroup analyses (Figure 4). The results showed that in the UA and NSTEMI subgroups, AIP was significantly associated with MACCE (both *P* < 0.05), whereas in the STEMI subgroup the association was not significant (*P* = 0.206). Across subgroups stratified by age, sex, BMI, LDL-C, and LVEF, elevated AIP was consistently associated with increased MACCE risk. No significant interaction was observed between AIP and any subgroup variable (all interaction *P* > 0.05).

### 3.5 Machine learning results

The correlation heatmap (Supplementary Figure S1) revealed significant collinearity among clopidogrel vs. ticagrelor use, stent number vs. stent length, and left main vs. target left main lesions. To avoid multicollinearity, ticagrelor use, stent number, and target left main lesion were excluded from the modeling. Then, the data were split in a ratio 7:3 into training (*n* = 506) and testing (*n* = 216) sets, with similar baseline characteristics (Supplementary Table S3).

**Figure 5:**
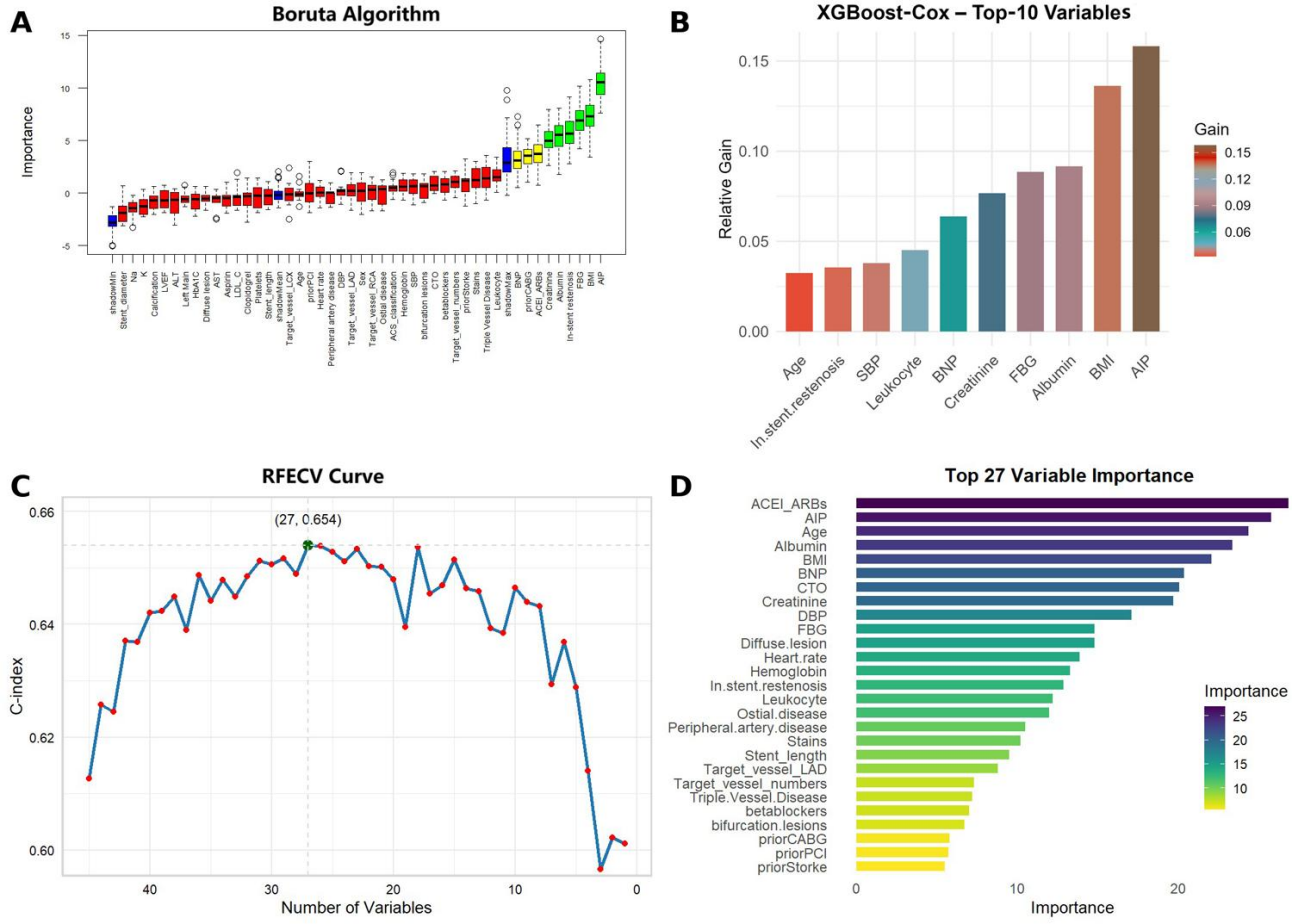
Feature selection results using boruta, XGBoost, and recursive feature elimination (RFE) algorithms. **(A)** Boruta algorithm selection results. Green indicates confirmed important features, yellow indicates tentative features, and red indicates unimportant features. **(B)** Top 10 features ranked by importance in XGBoost. **(C)** RFE method yielded the highest C-index with 27 variables. **(D)** Top 27 features ranked by importance from RFE.

**Figure 6:**
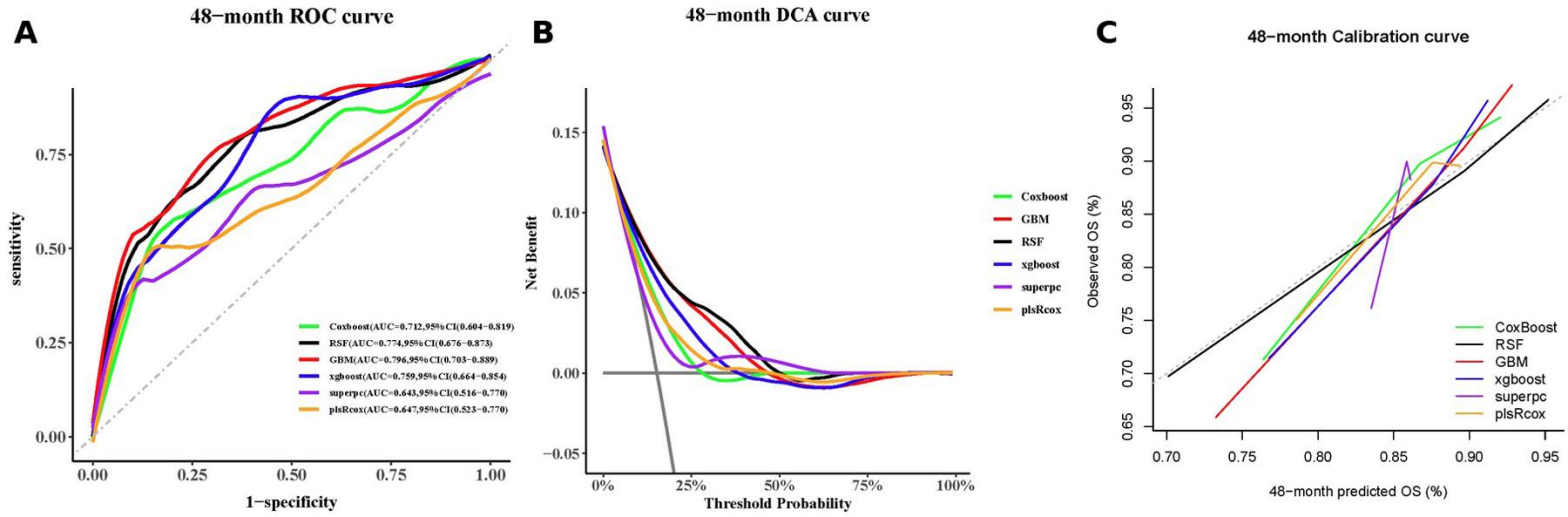
A 48-month performance of the machine-learning models: **(A)** receiver-operating-characteristic curves, **(B)** decision-curve analysis curves, and **(C)** calibration curves.

Using the Boruta algorithm for feature selection, important variables for predicting MACCE were identified (Figure 5A). Features marked green, yellow, and red were confirmed important, tentative, and unimportant, respectively. The XGBoost importance ranking shown in Figure 5B and Supplementary Table S4, highlighted the top 10 features. RFE results shown in Figures 5C,D, reveals the names of the top 27 features, which made the C-index to reach its maximum, when included. In all three methods, AIP was consistently identified as an important predictor. The final predictive model included the following variables: ACEI/ARBs, age, AIP, albumin, BMI, BNP, creatinine, FBG, in-stent restenosis, prior CABG, and leukocyte count. Six machine learning algorithms (CoxBoost, RSF, GBM, XGBoost, SuperPC, and plsRcox) were used to build the predictive models. The performances of each model at 12, 24, 36, 48 and 60 months are shown in Figure 6 and Supplementary Figures S2–S5. As AUC was predefined as the primary performance criterion, the GBM model achieved the best discrimination at 48 months, with the highest AUC of 0.796 (95% CI: 0.703–0.889) (Figure 6A). In addition, DCA and calibration curves, used as complementary assessments, suggested that the RSF model might provide the greatest net clinical benefit (Figure 6B) and showed good calibration, with its curve closely following the reference line (Figure 6C).

## 4 Discussion

To the best of our knowledge, this is the first study to assess the prognostic value of AIP in SMuRF-less patients with ACS. In a single-center cohort of 722 patients who underwent PCI and were followed up for a median of 59 months, elevated AIP levels were significantly associated with a higher risk of MACCE. Associations were also observed with all-cause mortality, non-fatal MI, and unplanned revascularization. A linear dose–response relationship between AIP and these adverse outcomes was confirmed by RCS analysis. Multivariable Cox regression further demonstrated that AIP remained an independent predictor of MACCE and its individual components after adjusting for confounding factors. In addition, machine learning models consistently ranked AIP as a key predictive feature, and the incorporation of AIP improved the overall predictive performance for MACCE.

SMuRF-less patients with ACS represent a distinct subgroup without traditional cardiovascular risk factors (2). Approximately 6.7% to 20.1% of ACS cases in recent studies are classified as SMuRF-less (30, 31). Previous studies reveal that SMuRF-less individuals with ACS may have worse clinical outcomes compared to their counterparts with one or more traditional risk factors. A multi-ethnic study involving 5,400 patients with ACS in Singapore reported 8.6% as SMuRF-less. Despite being younger, SMuRF-less patients have a higher likelihood of presenting with STEMI and experiencing markedly worse short-term outcomes, including increased 30-day all-cause and cardiac mortality and more frequent cardiogenic shock, compared to those with ≥1 SMuRFs (32). An analysis of 89,462 ACS cases from the Chinese CCC-ACS registry reveals that 11.0% of patients were SMuRF-less and had significantly greater in-hospital mortality than those with traditional risk factors (33). These findings suggest that SMuRF-lessness with ACS is often associated with rapid progression and more severe clinical manifestations, resulting in poorer short-term outcomes. Thus, the establishment of reliable prognostic markers is vital to facilitate early identification of high-risk individuals and guide timely, intensive therapeutic interventions.

AIP is a logarithmic index derived from the triglyceride/HDL-C ratio, reflecting the balance between atherogenic and protective lipoproteins. Previous studies show that AIP possesses predictive value for various cardiovascular conditions. In a retrospective observational study involving 404 patients with CCS, the relationship between AIP and major adverse cardiac events (MACE) was examined over a median follow-up of 35 months. During the study period, 88 patients experienced MACE, and the findings indicated that in patients with CCS, AIP was an independent predictor of long-term adverse clinical outcomes (11). Huang et al. also examined 2,250 patients with coronary artery disease undergoing PCI with drug-eluting stents to assess the association between AIP and target vessel revascularization (TVR). Over a median follow-up of 29.8 months, 106 (4.7%) patients developed TVR. A U-shaped relationship was observed between AIP and TVR risk, with a positive correlation when AIP exceeded 0.20 (34). Additionally, a study of 763 patients with STEMI undergoing primary PCI, found that higher AIP was independently associated with the no-reflow phenomenon, and that AIP outperformed triglyceride or HDL-C alone in predicting the no-reflow risk (35). Beyond cardiovascular outcomes, elevated AIP has also shown strong association with the incidence of hypertension, carotid atherosclerosis, and chronic kidney disease in cohort studies (12, 13, 36). Despite growing interest in AIP as a cardiovascular risk marker, its prognostic relevance in SMuRF-less patients with ACS remains unexplored. This study is the first to examine the predictive value of AIP for MACCE in a unique subset of patients. Moreover, we employed machine learning algorithms to build predictive models, and our findings consistently demonstrated that AIP holds predictive significance for adverse outcomes in this high-risk group.

The association between AIP and adverse outcomes likely reflects underlying metabolic and inflammatory dysregulation in SMuRF-less patients. Increasing evidence suggests that, in addition to traditional risk factors, multiple mechanisms continue to influence the development and progression of coronary heart disease (37–40). Among the most significant are IR and inflammatory processes.

IR contributes to metabolic dysregulation, characterized by the accumulation of glucose and free fatty acids in the circulation. Hyperglycemia directly triggers endothelial inflammation and impairs endothelial barrier function, while lipid peroxidation results in oxidized LDL, which deposits in the vascular intima, stimulates macrophages to transform into foam cells, and promotes fatty streak formation (41, 42). Additionally, IR suppresses the PI3K/Akt signaling pathway, leading to the buildup of reactive oxygen species (ROS) (43). These ROS damage mitochondrial function, enhance endothelial cell apoptosis, and impair vascular smooth muscle cell function, thereby accelerating atherosclerotic progression (44). Notably, these detrimental effects occur even in individuals without diabetes (45). Under IR conditions, an imbalance between inflammatory and insulin signaling can activate sterol regulatory element-binding protein-1c (SREBP-1c), increasing triglyceride levels (46). IR also causes a decline in HDL-C, primarily due to reduced levels of large HDL₂ particles and diminished lipoprotein lipase activity (47).

Inflammation has attracted increasing scientific interest as a key contributor to atherosclerotic cardiovascular disease. Accumulating evidence indicates that it plays a central role in multiple stages of atherosclerotic plaque progression, particularly in promoting plaque instability and triggering ACS (48, 49). Triglyceride-rich lipoproteins can be taken up by macrophages and converted into foam cells, which in turn promote inflammatory responses and atherosclerosis progression (50). Moreover, during inflammatory states, myeloperoxidase can mediate the oxidation of apolipoprotein A1, leading to reduced HDL-C function and concentration. This further exacerbates systemic inflammation and ultimately increases the risk of atherosclerosis (51).

As a lipid-derived index, AIP reflects the interplay between triglyceride and HDL-C levels. Prior studies have demonstrated a strong association between the triglyceride/HDL-C ratio and IR, as assessed by the homeostatic model, supporting its utility as a surrogate marker for IR (52). In addition, a high triglyceride/HDL-C ratio has been associated with elevated levels of inflammatory biomarkers. For example, a study involving patients with idiopathic pulmonary arterial hypertension found that a higher triglyceride/HDL-C ratio is significantly associated with increased levels of inflammatory markers, including interleukin-1β, monocyte chemoattractant protein-1, and interleukin-6, suggesting that the triglyceride/HDL-C ratio may also reflect systemic inflammatory status (53). In addition, emerging evidence indicates that AIP level is associated with conditions such as sleep apnea, genetic variations, and chronic infections (54–56). These findings suggest that AIP level may predict adverse outcomes in SMuRF-less populations by reflecting underlying mechanisms such as R and inflammation. Furthermore, compared with other indices of IR, AIP offers distinct advantages. Previous studies have shown that AIP often demonstrates stronger associations than other surrogate markers of IR (57). Moreover, AIP and the triglyceride-glucose index exhibit comparable performance in predicting the development and progression of coronary artery disease (58). However, AIP levels can be readily calculated from admission triglyceride and HDL-C levels, whereas triglyceride-glucose index and the metabolic score for IR rely on glucose, which is frequently influenced by stress hyperglycemia during the acute phase of ACS and prone to short-term fluctuations (59). Thus, AIP levels may serve as a simple and cost-effective tool in real-world clinical practice. It may help clinicians rapidly identify high-risk SMuRF-less patients whose proportion is underestimated by traditional risk scores, while a prediction model centered on AIP levels could further complement conventional tools and enable more efficient risk stratification. This, in turn, provides an opportunity to tailor follow-up strategies and consider earlier initiation of intensive therapies, such as lipid-lowering or anti-inflammatory treatment, thereby potentially improving this population prognosis.

### 4.1 Limitations

This study has several limitations. First, as a single-center retrospective cohort study, the findings may be subject to selection bias and potential residual confounding. Second, AIP level was assessed only at admission, and thus may not reflect its temporal variation, and could have influenced the prognosis. Future studies with longitudinal measurements or AIP trajectories are warranted. Third, part of the hyperlipidemia definition relied on lipid levels measured at admission, which may have been affected by the acute phase of ACS, leading to potential misclassification of the SMuRF-less status. Fourth, the number of SMuRF-less patients with ACS included in our study was relatively small. Fifth, given multiple endpoints and subgroup analyses, the risk of type I error cannot be excluded; therefore, our secondary or exploratory outcome findings should be interpreted with caution. Consequently, the findings should be interpreted with caution, and further validation in larger sample sizes and multi-center or independent cohorts is warranted to strengthen the robustness and generalizability of the results.

## 5 Conclusion

AIP, a readily accessible lipid-derived marker, demonstrates significant prognostic value in SMuRF-less patients with ACS. Elevated AIP levels are associated with increased risk of MACCE, highlighting its potential utility in identifying high-risk individuals lacking conventional cardiovascular risk factors, thus facilitating earlier clinical intervention.

## Data Availability

The raw data supporting the conclusions of this article will be made available by the authors, without undue reservation.
