# Supplementary material for: Prognostic value of the atherogenic index of plasma in patients with acute coronary syndrome without standard modifiable risk factors: a machine learning-based cohort study
Source: Front Cardiovasc Med. 2025 Oct 7;12:1681170. doi: 10.3389/fcvm.2025.1681170 (PMC12537706; doi:10.3389/fcvm.2025.1681170)
Supplement: Supplementary file 1 [file Datasheet1.docx]

Supplementary Material

**Supplementary Table 1** Missing data summary for baseline variables

| **Variables** | **Missing count (N)** | **Missing percentage (%)** |
| --- | --- | --- |
| SBP, mmHg | 3 | 0.4 |
| Leukocyte, x10^9^/L | 12 | 1.7 |
| AST, U/L | 7 | 1.0 |
| K, mmol/L | 7 | 1.0 |
| HbA1C, % | 13 | 1.8 |
| BNP, pg/mL | 21 | 2.9 |
| LVEF, % | 10 | 1.4 |

AST, aspartate transaminase; BNP, B-type natriuretic peptide; HbA1c, hemoglobin A1c; LVEF, left ventricular ejection fraction; SBP, systolic blood pressure

**Supplementary Table 2** Association between AIP tertiles and adverse outcomes

| **Outcomes** | **Overall** | **Tertile 1** | **Tertile 2** | **Tertile 3** | **P‑value** |
| --- | --- | --- | --- | --- | --- |
|  | **N = 722** | **n = 241** | **n = 240** | **n = 241** |  |
| MACCE | 168 (23.3) | 36 (14.9) | 55 (22.9) | 77 (32.0) | <0.001 |
| All-cause mortality | 27 (3.7) | 4(1.7) | 8 (3.3) | 15 (6.2) | 0.028 |
| Non-fatal myocardial infarction | 31 (4.3) | 5(2.1) | 9 (3.8) | 17 (7.1) | 0.023 |
| Unplanned revascularization | 129 (17.9) | 26 (10.8) | 43 (17.9) | 60 (24.9) | <0.001 |
| Non-fatal stroke | 25 (3.5) | 6 (2.5) | 8 (3.3) | 11 (4.6) | 0.456 |

AIP, atherogenic index of plasma; MACCE, major adverse cardiac and cerebrovascular events

**Supplementary Table 3** Baseline characteristics of the training and test sets

| Variables | Overall (N = 722) | Train (n = 506) | Test (n = 216) | P value |
| --- | --- | --- | --- | --- |
| MACCE, n (%) |  |  |  | 1 |
| 0 | 554 (76.7) | 388 (76.7) | 166 (76.9) |  |
| 1 | 168 (23.3) | 118 (23.3) | 50 (23.1) |  |
| Time, months | 55 (48, 60) | 56 (48, 61) | 55 (47, 60) | 0.428 |
| ACEI/ARBs, n (%) |  |  |  | 1 |
| 0 | 640 (88.6) | 449 (88.7) | 191 (88.4) |  |
| 1 | 82 (11.4) | 57 (11.3) | 25 (11.6) |  |
| Age, years | 60 (53, 67) | 60 (53.25, 67) | 62 (53, 67) | 0.728 |
| AIP | 0.14 ± 0.29 | 0.14 ± 0.28 | 0.13 ± 0.29 | 0.652 |
| Albumin, g/L | 43.0 ± 3.4 | 43.0 ± 3.5 | 42.9 ± 3.4 | 0.946 |
| BMI, kg/m^2^ | 25.5 (23.5, 27.6) | 25.5 (23.5, 27.6) | 25.7 (23.5, 27.7) | 0.855 |
| BNP, pg/mL | 47.5 (20, 94) | 46.5 (20, 94) | 48.5 (20, 89.3) | 0.562 |
| Creatinine, μmol/L | 71 (61.0, 80.5) | 70.8 (61, 81.5) | 71.6 (62.1, 79.0) | 0.793 |
| FBG, mmol/L | 5.39 (5.01, 5.92) | 5.39 (5.03, 5.92) | 5.38 (4.99, 5.87) | 0.262 |
| In-stent restenosis, n (%) |  |  |  | 0.132 |
| 0 | 698 (96.7) | 493 (97.4) | 205 (94.9) |  |
| 1 | 24 (3.3) | 13 (2.6) | 11 (5.1) |  |
| Prior CABG, n (%) |  |  |  |  |
| 0 | 709 (98.2) | 497 (98.2) | 212 (98.1) |  |
| 1 | 13 (1.8) | 9 (1.8) | 4 (1.9) |  |
| Leukocyte, x10^9^/L | 6.70(5.66, 8.02) | 6.62 (5.46, 8.03) | 6.85 (5.89, 7.99) | 0.079 |

ACEI, angiotensin converting enzyme inhibitor; AIP, atherogenic index of plasma; ARB, angiotensin II receptor blocker; BMI, body mass index; BNP, B-type natriuretic peptide; CABG, coronary artery bypass grafting; FBG, fasting blood glucose; MACCE, major adverse cardiac and cerebrovascular events.

**Supplementary Table 4** XGBoost Variables Importance

| Variables | Gain | Cover | Frequency |
| --- | --- | --- | --- |
| AIP | 0.158203 | 0.171882 | 0.111111 |
| BMI | 0.136306 | 0.193946 | 0.111111 |
| Albumin | 0.091695 | 0.086865 | 0.074074 |
| FBG | 0.088527 | 0.108541 | 0.088319 |
| Creatinine | 0.076673 | 0.090745 | 0.091168 |
| BNP | 0.063867 | 0.045605 | 0.062678 |
| Leukocyte | 0.045162 | 0.041215 | 0.045584 |
| SBP | 0.037918 | 0.01597 | 0.039886 |
| In-stent restenosis | 0.035585 | 0.073703 | 0.034188 |
| Age | 0.032513 | 0.016128 | 0.034188 |
| LVEF | 0.032334 | 0.008412 | 0.034188 |
| Stent length | 0.028253 | 0.012858 | 0.039886 |
| LDL-C | 0.026898 | 0.017596 | 0.039886 |
| Target vessel numbers | 0.023177 | 0.035683 | 0.034188 |
| ALT | 0.021188 | 0.025269 | 0.031339 |
| ACEI/ARBs | 0.016686 | 0.006423 | 0.017094 |
| Platelets | 0.015612 | 0.006696 | 0.011396 |
| Triple Vessel Disease | 0.013198 | 0.002665 | 0.011396 |
| Hemoglobin | 0.011544 | 0.014499 | 0.019943 |
| Heart rate | 0.007852 | 0.006244 | 0.008547 |
| Prior PCI | 0.006642 | 0.005972 | 0.011396 |
| DBP | 0.006181 | 0.002292 | 0.008547 |
| Beta blockers | 0.004671 | 0.000475 | 0.008547 |
| ACS classification | 0.004151 | 0.007078 | 0.008547 |
| AST | 0.004093 | 0.00034 | 0.008547 |
| HbA1C | 0.003902 | 0.001028 | 0.005698 |
| Sex | 0.003164 | 0.001396 | 0.002849 |
| Stent diameter | 0.002248 | 0.000196 | 0.002849 |
| Diffuse lesion | 0.001757 | 0.000279 | 0.002849 |

ACEI, angiotensin converting enzyme inhibitor; ACS, acute coronary syndrome; AIP, atherogenic index of plasma; ALT, alanine transaminase; ARB, angiotensin II receptor blocker; AST, aspartate transaminase; BMI, body mass index; BNP, B-type natriuretic peptide; DBP, diastolic blood pressure; FBG, fasting blood glucose; HbA1c, hemoglobin A1c; LDL-C, low-density lipoprotein cholesterol; LVEF, left ventricular ejection fraction; PCI, percutaneous coronary intervention; SBP, systolic blood pressure.


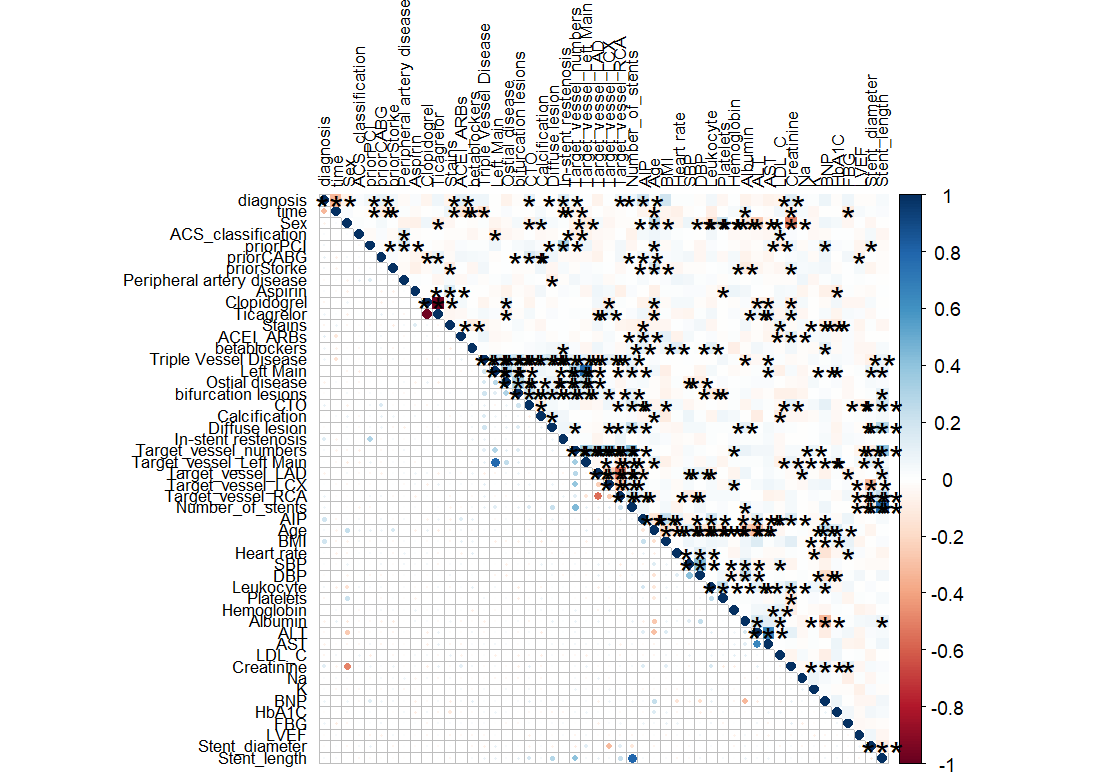


**Supplementary Figure 1.** The correlation heatmap


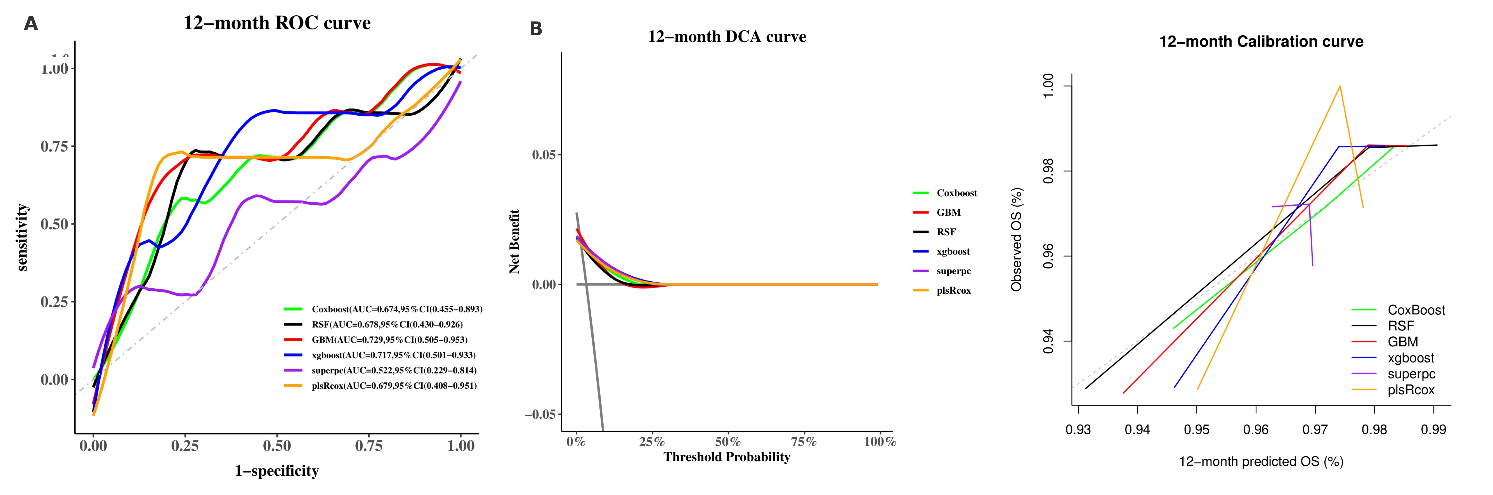


**Supplementary Figure 2.** The 12-month performance of the machine-learning models: (A) receiver-operating-characteristic curves, (B) decision-curve analysis curves, and (C) calibration curves.


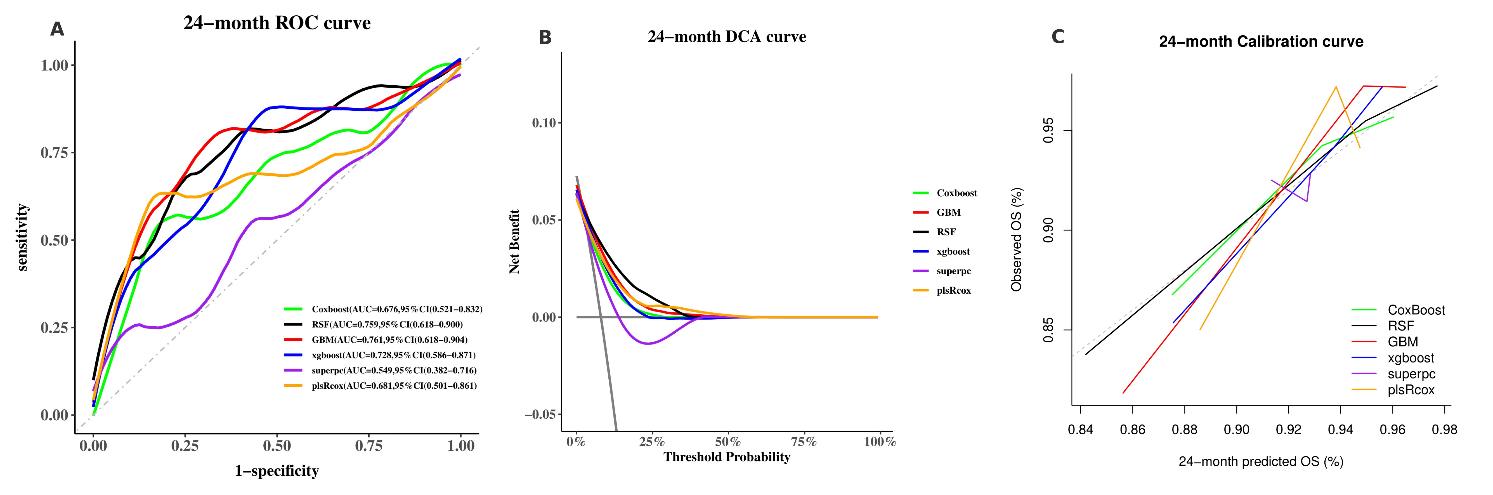


**Supplementary Figure 3.** The 24-month performance of the machine-learning models: (A) receiver-operating-characteristic curves, (B) decision-curve analysis curves, and (C) calibration curves.


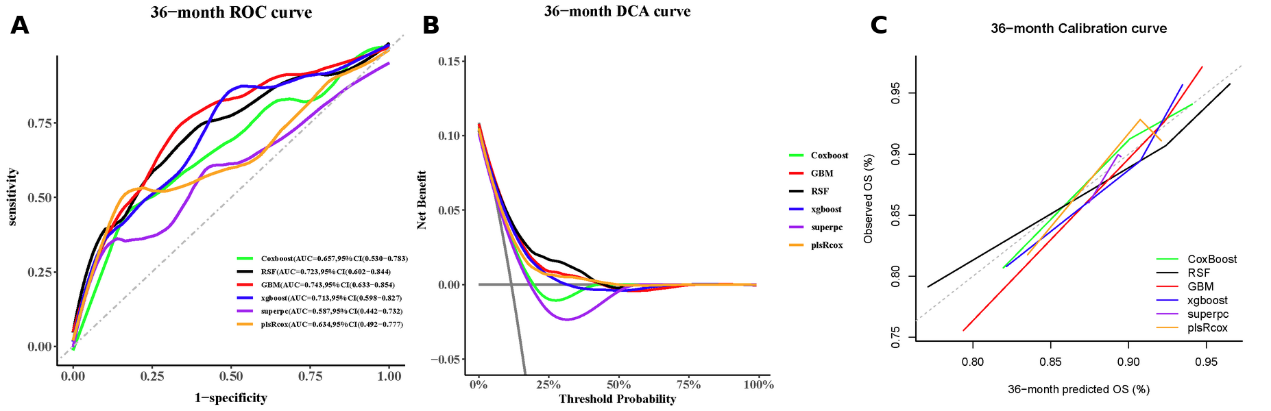


**Supplementary Figure 4.** The 36-month performance of the machine-learning models: (A) receiver-operating-characteristic curves, (B) decision-curve analysis curves, and (C) calibration curves.


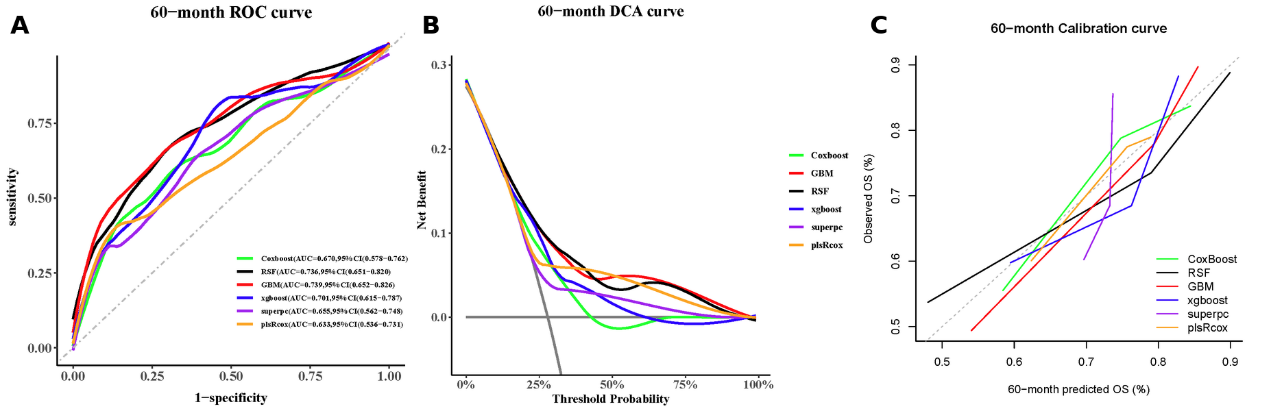


**Supplementary Figure 5.** The 60-month performance of the machine-learning models: (A) receiver-operating-characteristic curves, (B) decision-curve analysis curves, and (C) calibration curves.

.
